# Supplementary material for: HacDivSel: Two new methods (haplotype-based and outlier-based) for the detection of divergent selection in pairs of populations
Source: PLoS One. 2017 Apr 19;12(4):e0175944. doi: 10.1371/journal.pone.0175944 (PMC5397020; doi:10.1371/journal.pone.0175944)
Supplement: S1 Appendix — (DOCX) [file pone.0175944.s001.docx]

**Supplementary Appendix.**

**HacDivSel: Two new methods (haplotype-based and outlier-based) for the detection of divergent selection in pairs of populations.**

Antonio Carvajal-Rodríguez ^1*^

^1^ Departamento de Bioquímica, Genética e Inmunología. Universidad de Vigo, 36310 Vigo, Spain.

**Keywords:** haplotype allelic class, *F*_ST_, *G*_ST_, outlier test, divergent selection, genome scan, non-model species.

**:* Corresponding author

Email: [acraaj@uvigo.es](mailto:acraaj@uvigo.es)

# Appendix

**A-1) Effect of window size on gSvd**

We can appreciate the effect of a window size *L* on the computation of the original *gSvd* measure as follows. Recall that the HAC distance *d* between an haplotype *h* and a reference *R* both of length *L* is

$d= \sum_{i=1}^{L} I\left( h_{i}\neq R_{i} \right)$

where *I*(A) is the indicator function of the event *A*. Thus, *d* ∈[0, *L*] so that, given an increase of the window size by *Q* (*Q* > 1), then *d* ∈[0, Q*L*]. Therefore, the change in window size is a change in the scale of the HAC distances. Depending on the distribution under the new window size the magnitude of the change in the scale can be *Q* or more generally *Q*' ∈(1, *Q*]. Thus, a window size increase of *Q* has a quadratic impact onto *S*^2^ and Δ as defined in (1). Then, if we define *gSvd* for a window size *L*_A_, we have

${gSvd}_{i}= \frac{S_{2i}^{2}-S_{1i}^{2}}{L_{A}}\times f_{i}{(1-f_{i})}^{a}\times b$

and if we change to window size *L*_B_ = *QL*_A_ we might have

*gSvd_LB_* = *QgSvd_LA_*

For the equation to be exact it is also necessary that the change of window size do not alter the frequency distribution so that the relationship *v*_B_ = *Q*^2^*v*_A_ and Δ_B_ = *Q*^2^Δ_A_ holds on, if not, the change will be better defined by *Q*' ∈(1, *Q*]. In any case, increasing the window size by *Q* may also provoke a proportional increase of the statistic. This explains why the normalization, dividing by *L*, performed within gSvd is not very effective on avoiding a systematic increase of the statistic under higher window sizes (Hussin et al. 2010; Rivas, Dominguez-Garcia, and Carvajal-Rodriguez 2015).

**A-2) Normalized variance difference**

Consider the frequencies of a given haplotype *h* within each partition 1 and 2 having *n*_1_ and *n*_2_ total number of haplotypes respectively and *n* = *n*_1_ + *n*_2_

$f_{h1}= \frac{n_{h1}}{n_{1}}$ $f_{h2}= \frac{n_{h2}}{n_{2}}$ $f_{h}= \frac{n_{h1}+n_{h2}}{n}=\frac{{f_{h1}n}_{1}+f_{h2}n_{2}}{n}$ (A-2-1)

Let *d*_h_ be the HAC distances for each haplotype *h* and with some abuse of notation *F*, *F*_1_, *F*_2_ the frequency distribution in the whole sample and in the partitions *P*_1_ and *P*_2_ respectively.

$$m= \sum_{h}^{n} \frac{d_{h}}{n}= \sum^{F} d_{h}f_{h}=\sum^{F} d_{h}\frac{{f_{h1}n}_{1}+f_{h2}n_{2}}{n}= \sum^{F1} d_{h}\frac{{f_{h1}n}_{1}}{n}+ \sum^{F2} d_{h}\frac{{f_{h2}n}_{2}}{n}$$

Note that

$m_{1}=\sum^{F1} d_{h}f_{h1}$ and $m_{2}=\sum^{F2} d_{h}f_{h2}$ and then

$m= \frac{n_{1}m_{1}+n_{2}m_{2}}{n}$ (A-2-2)

Now consider the variance

$v= \sum^{F} {{(d}_{h}- m)}^{2}f_{h}=\sum^{F} d_{h}^{2}f_{h}- m^{2}$ (A-2-3)

$v_{1}= \sum^{F1} d_{h}^{2}f_{h1}-m_{1}^{2}$ $v_{2}= \sum^{F2} d_{h}^{2}f_{h2}-m_{2}^{2}$.

Substituting (A-2-1) in (A-2-3) and after some rearrangement we finally get

$v-\bar{V}= \Delta$ (A-2-4), with

$\bar{V}= \frac{n_{1}v_{1}{+ n}_{2}v_{2}}{n}$and $\Delta=\frac{n_{1}n_{2}}{n^{2}}{(m_{1}-m_{2})}^{2}=\frac{n_{1}n_{2}}{n^{2}} \Delta_{m}^{2}$.

where *v*_1_ and *v*_2_ are the variances at each partition,

Note that max(Δ) = *L*^2^/4 = max(*v*) and min(Δ) = 0.

If we consider the sampling variance *S*^2^ instead of the variance we have similarly

$S^{2}-\bar{S}= \frac{n}{n-1}\Delta$ (A-2-5)

being $\bar{S}= \frac{{(n}_{1}-1)S_{1}^{2}{+(n}_{2}-1)S_{2}^{2}}{n-1}$.

From (A-2-5) and defining *k* as the fraction of sequences in the minor partition then *n*_1_ = (1-*k*)*n* and *n*_2_ = *kn* with *k* ∈(MAF, 0.5) if *n*_1_ ≥ *n*_2_ or *k* ∈(0.5, 1-MAF) if *n*_1_ ≤ *n*_2_. Then we can express *S*^2^ as

$S^{2}=\frac{\left[ \left( 1-k \right)n-1 \right]S_{1}^{2}+ (k{n-1)S}_{2}^{2}}{n-1}+ \frac{n\left( 1-k \right)k}{n-1}\Delta_{m}^{2}$ and

$S_{2}^{2}=\frac{{(n-1)S}^{2}-\left[ \left( 1-k \right)n-1 \right]S_{1}^{2}-n\left( 1-k \right)k\Delta_{m}^{2}}{kn-1}$

So that the variance difference can be broken down in two terms

$S_{2}^{2}- S_{1}^{2}=\frac{{(n-1)S}^{2}-(n-2)S_{1}^{2}}{kn-1}- \frac{n\left( 1-k \right)k\Delta_{m}^{2}}{kn-1}$

Reordering we have

$\frac{S_{2}^{2}- S_{1}^{2}}{\left( 1-k \right)k}=\frac{{(n-1)S}^{2}-(n-2)S_{1}^{2}}{(kn-1)\left( 1-k \right)k}- \frac{n\Delta_{m}^{2}}{kn-1}$ (A-2-6)

Realize that the first term in the sum is contributing to increase the variance difference whenever (n-1)*S*^2^ ≥ (n-2)S_1_^2^. Note also that (1-*k*)*k* in the denominator has it maximum value when *k* = 0.5. The second term in the sum, _m_^2^ increases with directional selection (*m*_1_ => 0 because the haplotypes in *P*_1_ are expected, by definition, to be closer to the reference configuration) while *kn* (= *n*_2_) decreases, so, both are contributing to increase the negative term under selection and diminish the value of the statistic. Thus, because our aim is to increase the value of the statistic in the presence of selection it is convenient to discard the second term in the variance difference (A-2-6). Now, recall that the generalized Svd defined for any SNP *i* is

${gSvd}_{i}= \frac{S_{2i}^{2}-S_{1i}^{2}}{L}\times f_{i}{(1-f_{i})}^{a}\times b$

and, after discarding the second term in (A-2-6), substituting it in the (*S*^2^_2i_ - *S*^2^_1i_)/*L* term in gSvd_i_ and taking *a* = 1 and *b* = 4 we obtain

${vd}_{i}= \frac{(n-1)S^{2}-(n-2)S_{1i}^{2}}{kn-1}\times{4f}_{i}(1-f_{i})$ (A-2-7)

We can appreciate that decreasing *S*_1_ and increasing *S*_2_ will increase the value of the statistic (because *S*_2_ increases *S*). If *S*_1_ and *S*_2_ are equal to, say S_x_, then we have

$\bar{S}= \frac{{(n}_{1}-1)S_{1i}^{2}{+(n}_{2}-1)S_{2i}^{2}}{n-1}= \frac{(n-2)S_{\mathrm{xi}}^{2}}{n-1}$

$S^{2}=\frac{({n-2)S}_{\mathrm{xi}}^{2}}{n-1}+ \frac{n}{n-1}\Delta$

$\left( n-1 \right)S^{2}-\left( n-2 \right)S_{\mathrm{xi}}^{2}=n\Delta$

${vd}_{i0}= \frac{n\Delta}{n_{2}-1}\times{4f}_{i}(1-f_{i})$ =${4{[f}_{i}(1-f_{i})]}^{2}\times\frac{n{(m_{1}-m_{2})}^{2}}{n_{2}-1}$

which is independent of the variances and just relies on the partitions’ size means (*m*_1_ and *m*_2_) and on the candidate SNP frequency. This term appears because of the value that has been discarded in (A-2-7) multiplied by the SNP frequency. Note that we can express (A-2-7) as

${vd}_{i}= {vd}_{i0}+{4f}_{i}\left( 1-f_{i} \right)({S_{2i}^{2}-S}_{1i}^{2})$ (A-2-8)

corresponding to formula (2) in the main text.

Thus, the effect of selection upon *vd*_i_ is two-fold. By one side, for a given value of *m* in the sample, it decreases the value of *m*_1_ and so increases *vd*_i_. By the other side, for a given variance in the non-selective partition, the effect of selection diminishes the variance *S*_1_^2^ in the selective partition also increasing *vd*_i_.

It is worth mentioning that the two parts of *vd*_i_ are not independent (recall that the HAC values are bounded by 0 and *L*). So that, having an extreme value for the HAC mean in the selective partition, say *m*_1_ = 0, this implies that *S*_1_^2^ = 0 since every haplotype has to have a HAC of 0 to get that mean value. Note however that the opposite is not true, a value of *S*_1_^2^ = 0 does not imply necessarily that *m*_1_ = 0.

*Upper bounds*

We want to know what would be the value of *vd*_i_ when one of the variances is 0 and the other is at the upper bound.

Thus, *S*_1_^2^ =0 and *S*_2_^2^ = *maxS*_2_^2^ then

${vd}_{i{(s}_{1}=0,s_{2}=\max)}= {vd}_{i0maxS_{2}}+{4f}_{i}\left( 1-f_{i} \right){maxS}_{2}^{2}$

the range in the partition 2 is *L* -1 so that an upper bound for *S*_2_^2^ is (Sharma, Gupta, and Kapoor 2010)

$\max S_{2}^{2}\leq\frac{n_{2}}{n_{2}-1}\frac{{(L-1)}^{2}}{4}$

The bound can be reached only when half of the HAC values in the partition 2 are *L* and the others are 1 so that *m*_2_ = (*L*+1) / 2 and

${vd}_{i0maxS_{2}}= 4\frac{{{[f}_{i}(1-f_{i})]}^{2}n}{n_{2}-1}{(m_{1}-\frac{L}{2}- \frac{1}{2})}^{2}$

since we already assumed that the variance in the first partition is 0 we can maximize the difference by considering that *m*_1_ = 0 then we get

${vd}_{i0maxS_{2}}= \frac{{{[f}_{i}(1-f_{i})]}^{2}n}{(n_{2}-1)}{(L+1)}^{2}$

By noting that *n*_2_ = *f*_i_*n* it is possible to show that the derivative with respect to *f*_i_ is not null at *f*_i_ = 0.5 so this point (*n*_2_ = *n*/2) is not a maximum.

Instead of maximizing the variance difference we could alternatively maximize the component for the difference between the means. This occurs when *m*_1_ = 0 and *m*_2_= *L* so that (*m*_1_ - *m*_2_)^2^ = *L*^2^. In this case the variances at each partition are zero (the HAC is 0 in all haplotypes in partition 1 and is *L* in all haplotypes in the partition 2) so, (A–2–8) becomes

${vd}_{i0max}= H^{2}\times\frac{nL^{2}}{f_{i}n-1}$ (A–2–9)

where *H* = 2*f*_i_(1 - *f*_i_) and *f*_i_*n* = *n*_2_.

The value of *vd*_i0max_ is always higher than *vd*_i0maxS2_. However, the absolute maximum of *vd*_i0max_ depends on the frequencies and does not occur at intermediate values. We are interested in first, a quantity independent of the frequencies and second, that be a maximum when the frequencies are intermediate. The value of (A–2–9) when substituting by intermediate frequency is

${vd}_{i0max}(f_{i}=0.5)=\frac{nL^{2}}{2(n-2)}=d_{max}$ (A-2-10)

which is an upper bound of (A-2-8) at intermediate frequencies.

We can still look for another upper bound considering the variance in the whole sample while forgetting the variance within the partitions. In this case the range is from 0 to *L* and we get immediately

$S_{\max}^{2}\leq\frac{n}{(n-1)}\frac{L^{2}}{4}$

Again, for reaching the bound it is necessary that half of the values be 0 and the other half be *L* which in turn implies intermediate frequencies and null variances within the partitions (remember that HAC=0 is only possible in the partition 1 while HAC = *L* only in the partition 2). Therefore, we should expect that when using this bound the variance difference coincide with (A-2-10). To check that, if we substitute in (A-2-7) with the upper bound for *S*^2^we get

${vd}_{i(S_{max}^{2})}= \frac{2(n-1)S^{2}}{n-2}=\frac{2(n-1)nL^{2}}{4(n-1)(n-2)}=d_{max}$

as expected.

*Normalizing the variance difference*

As we saw, the quantity *d*_max_ is an upper bound of equation (A-2-8) when the frequencies are intermediate. Then we may normalize the variance difference dividing by this quantity

${nvd}_{i}= \frac{{vd}_{i0}+{4f}_{i}\left( 1-f_{i} \right)({S_{2}^{2}-S}_{1}^{2})}{d_{max}}$

The motif for using *d*_max_ instead the bound from (A–2–9) is because we are not interested in that the bound varies with the frequencies. We focus only in the highest *nvd* that correspond with intermediate frequencies. If there are other high *nvd* values that are not at intermediate frequencies they will be discarded by the *F*_ST_ part of the test.

*Neutral distribution*

We performed neutral coalescent simulations using the ms program (Hudson 2002) to simulate neutral samples (n=50) with 100,000 segregating sites from two populations connected by migration (*Nm*=10) with recombination (ρ=120). In Fig A we can appreciate the *nvd* distribution of 60,000 shared segregating sites. Each panel correspond to a different window size *L*.


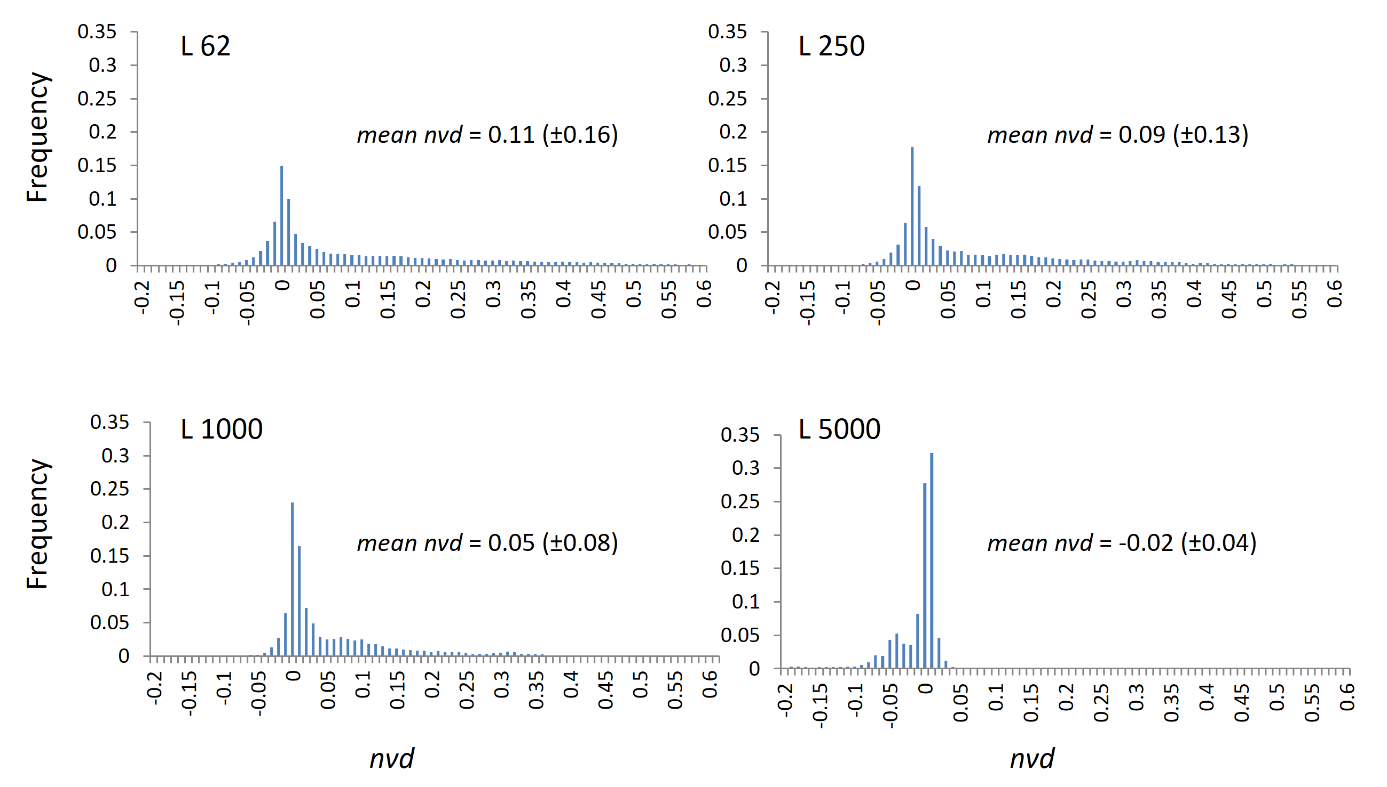


Fig A. Neutral distribution of the *nvd* statistic for samples with 60,000 segregating sites (ρ = 120) under different window sizes *L*.

By comparing the distribution at different windows it is clear that the effect of increasing the window size is a slight reduction of the *nvd* mean value and variance. The higher the window size the more the distribution is displaced to the left and is more centered on zero.

**A-3) Lower bound of *nvd* and sign test**

Now we consider the maximum value *S*_1_*^2^*_max_ for the variance in the first partition. If the candidate gene is at intermediate frequencies then 4*f*(1-*f*) would be close to 1, *n*_1_ = *n*_2_ = *n*/2 and by substituting in (A-2-7) *S*_1_*^2^* by *S*_1_*^2^*_max_ then (*n*-2) *S*_1_*^2^*_max_ = *n*(*L*-1*)*^2^/4. Note that in this case the value *m*_1_ is fixed to (*L*-1)/2 so finally we get

$LB{nvd}_{i}=\frac{4\left( n-1 \right)S^{2}-n({L-1)}^{2}}{nL^{2}}$ (A-3-1)

which is a lower bound for *nvd* under a given *S*^2^. Note that the variance in the first partition should not be at its maximum if selection is acting. Therefore a value as low as in (A-3-1) is not expected under selection. The lower bound still depends on the variance in the second partition and on the absolute value of the difference between the partition’s means |*m*_1_ - *m*_2_|. If the variance in the second partition is maximum it will be equal to the variance in the first and (A-3-1) becomes *vd*_i0_ divided by *d*_max_ and then, it becomes

$LB{nvd}_{i0}= \frac{\Delta_{m}^{2}}{L^{2}}=\frac{1}{L^{2}}$

On the contrary, for any window size higher than 6, if the variance in the first partition is the maximum and in the second partition is zero then the lower bound would be negative independently of the value *m*_2_. Also, if |*m*_1_ - *m*_2_| is low, then the variance in the second partition cannot reach its upper bound, and again, the lower bound would be negative.

That is, with small variance in the second partition or when |*m*_1_ - *m*_2_| is low, just like should be expected under neutrality, the lower bound is negative. Note that, if *n*_1_ = *n*/2, (A-3-1) is equal or lower than

$\frac{4\left( n-1 \right)S^{2}-2\sum_{i} \mathrm{hac}_{1i}^{2}}{nL^{2}}$ (A-3-2)

where hac_1i_ are the HAC values measured at each haplotype *i* in the partition 1 and the sum is over the *n*_1_ sequences in that partition. However if *n*_1_ > *n*/2, the quantity in (A-3-2) could be higher or lower than (A-3-1) depending on the HAC values of the first partition. Recall that, if the SNP that performs the partition is under selection, we expect low values of *m*_1_ (close to the reference haplotype). Therefore, if it happens that (A–3–2) is negative when the frequencies are intermediate this is not expected under divergent selection. In any case, a negative value in (A-3-2) may be caused by *m*_1_ being equal or higher than *m*_2_ and suggests that the value of *nvd* is not the result of divergent selection. Indeed, we call (A-3-2) the selection sign (*ssig*, formula 5 in the main text) and require it to be positive to count a given candidate as significant.

In Fig B we observe the distribution of the maximum *nvd* value compared among neutral and selective scenarios and with the reference haplotype computed from population 1 or 2. There is no effect of using one reference or another under the neutral scenarios while the impact is almost unappreciable under the selective scenarios (see also Table A). On the contrary, the selective and neutral distributions are quite different. With the mean maximum *nvd* and variance being almost 1 order of magnitude higher in the selective cases. The mean selection sign is negative only under the neutral scenario.


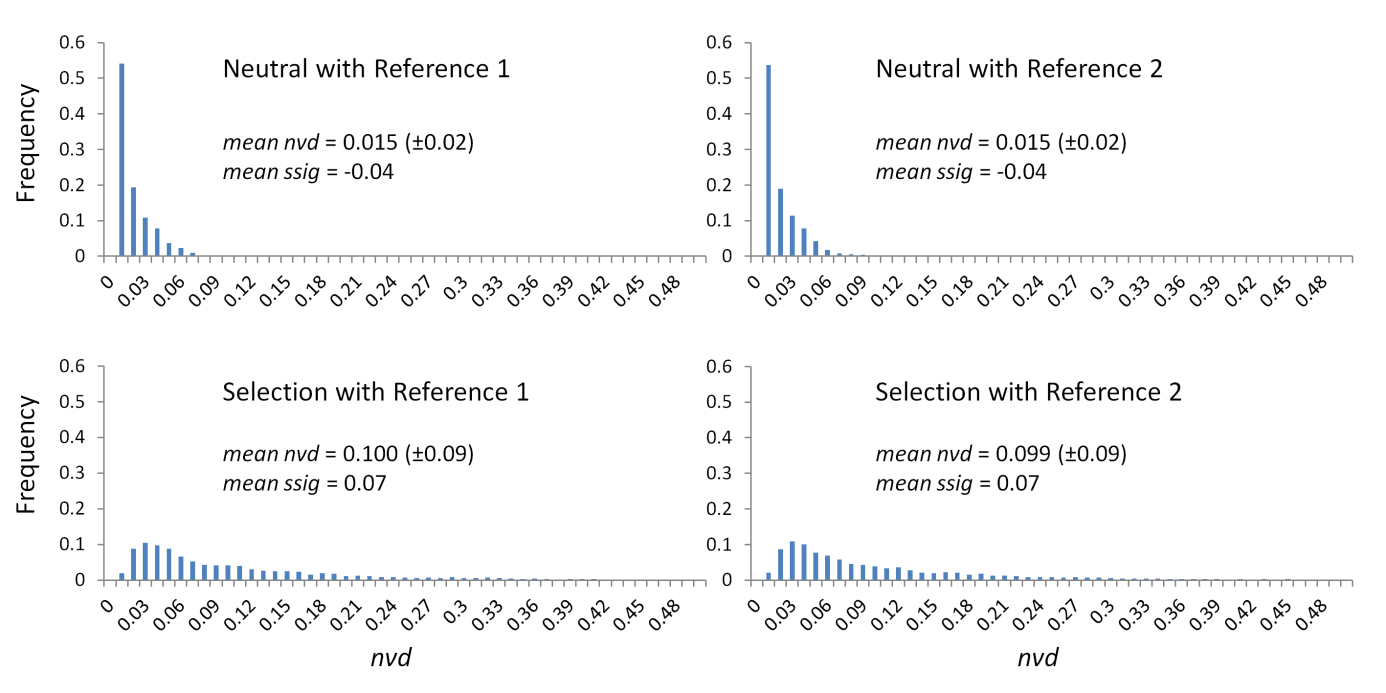


Fig B. Comparison of the distribution of maximum *nvd* values for neutral and selective cases computed using as reference the first (Reference 1) or the second (Reference 2) population. The maximum *nvd* was computed for each of 1,000 independent runs of about 250 segregating sites (ρ = 60) under 3 different window sizes (a total of 3,000 values).

In Fig B we have compared the distribution of the maximum *nvd* value using as reference one population or another for the case of recombination rate ρ=60. In Table A we can see the same comparison in terms of power and false positive rate separated for different recombination rate and window size. The false positive rate is the same whatever the reference and the power is very similar with at worst a 5% difference in power.

**Table A. *Nvd* power and false positive rate (% of runs) obtained when setting the reference haplotype from population 1 (Ref 1) or from population 2 (Ref). The different cases correspond to mutation θ=60 with different recombination (ρ) values.**

| **Case θ, ρ** | **Average window size** | **% Power (% false)** | |
| --- | --- | --- | --- |
|  |  | Reference 1 | Reference 2 |
| 60, 0 | 251 | 77 (1) | 76 (1) |
|  | 135 | 80 (1.5) | 80 (1.5) |
|  | 76 | 84 (1) | 84 (1) |
|  |  |  |  |
| 60, 4 | 232 | 87 (2) | 83 (2) |
|  | 123 | 87 (4) | 87 (4) |
|  | 70 | 89 (5) | 86 (5) |
|  |  |  |  |
| 60, 60 | 249 | 43 (0) | 38 (0) |
|  | 124 | 81 (0.1) | 77 (0.2) |
|  | 63 | 88 (3) | 88 (3) |
|  |  |  |  |

**A-4) Bounds on FDR and q-value estimation**

For a given test *i* with *p*-value *p*_i_ the FDR after performing *S* tests is just (Storey 2002; Storey and Tibshirani 2003)

FDR(*p*_i_) = *p*_i_ *π_0_*S/*max*(#{*p* ≤ *p*_i_} ,1)

where π_0_ is the proportion of true nulls and #{*p* < *p*_i_} corresponds to the position of *p*_i_ in the sorted (in ascending order) list of *p*-values. Then the *q*-value for *p*_i_ is obtained as the minimum of the FDR set for the *p*-values equal or higher to *p*_i_.

*q*(*p*_i_) = *min*{FDR(*t*)} with *t* ≥ *p*_i_.

We estimate π_1,_ i.e. the proportion of the false null hypothesis using a method that is specially aided for cases when this proportion is very small (Meinshausen and Rice 2006) and then we obtain π_0_ = 1 - π_1_. This is adequate given our expectation of detecting some few positions in the genome belonging to the alternative non-neutral distribution.

*Lower bound*

In the EOS test and because we have a sample-dependent upper bound *G*^*^_STmax_ for the *G*_ST_ estimator we can correct for the minimum *q*-value achievable in that sample. Then for a given sample with *G*_ST_ mean *m,* the lower-bound of the *p*-value for the *G*_ST_ test *f* will be a function *p*_LB_ = *f*(*G*^*^_STmax_,*m*) ≥ 0 that can be computed for each sample and consequently we can guess a minimum *q*-value. Therefore for the lower bound *p*-value *p*_LB_, we have

FDR(*p*_LB_) = *p*_LB_ *π_0_**S*/*max*(#{*p* ≤ *p*_LB_} ,1) ≤ *p*_LB_ *π_0_**S* so that a lower-bound FDR is

FDR_LB_ = *p*_LB_ *π_0_**S*/*max*(#{*p* ≤ *α*} ,1) < FDR(*p*_LB_)

since #{*p*≤ *p*_LB_} < #{*p* ≤ *α*} and provided that *p*_LB_ < α.

Note that with π_0_ < 1 i.e. some non-null may exist, and because *p*_LB_ is above zero then we cannot reach a null FDR neither in the case when the lowest *p*-value corresponds to the true effect.

Now, let

*q*(*p*_LB_) = *min*{FDR(*t*)} with *t* ≥ *p*_LB_ then a lower bound for the *q*-value is

q_LB_ = *min*{ *q*(*p*_LB_), FDR_LB_ }.

*Upper bound*

Let *p*_i_ = α then we get FDR(α) = α*π_0_**S*/*max*(#{*p* ≤ α } ,1). Therefore, *q*(α) = *min*{FDR(*t*)} with *t* ≥ α is the minimum FDR that can be committed when calling significant a given test at this threshold. If the *p*-values are uniformly distributed, the expected *q*(α) is π_0_. However, if the *p*-value distribution is weighted towards 0, as expected when we have a mixture of null and alternative distributions, then *q*(α) < π_0_. Now, if we set a threshold of 1 i.e. the whole distribution of values, then we get necessarily a FDR equal to π_0_ thus

*q*(1) = FDR(1) = 1 *π_0_*S/S = π_0_ and π_0_ ≤ 1 so that the upper bound is *q*_UB_ = 1.

*Corrected q value*

Under some circumstances may be of interest to correct for the bias generated by not being able of reaching minimum *p*-values due to the sample-dependent *G*_ST_ upper-bound. Thus we define

*q'*(*p*_i_) = (*q*(*p*_i_) - *q*_LB_) / (1 - *q*_LB_)

*Dependence and q-values*

When considering many SNPs through the genome, the condition of independence is rarely maintained. In general, FDR-based estimates become more conservative as the dependence is stronger (Storey 2001; Storey, Taylor, and Siegmund 2004; Friguet 2012). An important aspect when computing the FDR and the associated *q*-values, is the estimation of the proportion of true null hypotheses, π_0_. As indicated above we estimate π_0_ through π_1_. In our case, the impact of dependence structures on the estimation of π_1_ has proved to be negligible compared to the conservative impact of the dependence on the FDR estimation. This is not surprising because most SNPs belong to the true null distribution so we do not expect the density of correlated values to be weighted towards 0. Thus, on the contrary, for *p*_i_ sufficiently low, it should be true that #{*p* ≤ *p*_i_} < *Sp*_i_ i.e. FDR(*p*_i_) > π_0_. This means that we tend to have conservative estimates of FDR. We have confirmed this when comparing *q*-value estimates from dependent versus independent data. For example, when comparing *q*-values for the EOS test in files with linked SNPs (ρ = 60; 1.5 cM/Mb) versus files with non-linked SNPs, all other being equal, we obtained *q*=*q*'= 2.4 x 10^-6^ on average when markers are independent versus *q*= 0.63, *q*' = 0.5 when each pair of markers are linked.

**A-5) Lower and upper bounds for *G*_ST_ and *F*_ST_ estimators**

Let *Np* > 1, be the number of populations, *na* is the number of alleles, 1 - *maf* is the major allele frequency and *n*_i_ is the sample size for population *i*. Note that the alleles can be different in different populations and that, without loss of generality, we assume that the number *na* of alleles is the same in every population.

*a) G_STmax_*

For *G_ST_* (Nei 1973) we develop the formulas just for the one locus case as this simplifies the notation and does not imply loss of generality. We will obtain the maximum *G*_ST_ noted as *G*_STmax_ and an upper bound noted as

*G*^*^_STmax_ = 1 – *H*^*^_smin_/*H*^*^_Tmax_ (A-5-1)

Where

$$H_{\mathrm{smin}}^{*} =1- \left( 1-maf \right)^{2}-\left( maf \right)^{2}$$

and

$$H_{Tmax}^{*}=H_{\mathrm{smin}}^{*}+\left( 1-maf \right)^{2}\left( \frac{Np -1}{Np} \right)+\left( maf \right)^{2}\left( \frac{Np -1}{Np} \right)$$

Let *G*_ST_ = 1 – *H*_s_/*H*_t_ with *H*_s_ = 1 – $\sum_{i=1}^{na} p_{i}^{2}$ averaged for the different populations and *H*_t_ is the same computation as *H*_s_ but performed with the pooled metapopulation allele frequencies (Charlesworth and Charlesworth 2010). We are interested in computing the maximum *G*_ST_ when the major allele frequency (1 - *maf*) is not 1. Additionally, we want to show that *G*^*^_STmax_ is an upper bound of such value independently of the number of alleles considered. In doing so, we first compute the minimum for the subpopulation heterozigosity, then we compute the maximum for the pooled heterozygosity and subsequently we use these two values for computing the maximum *G*_ST_. This maximum will depend on the number of alleles segregating at each population. Finally we demonstrate that (A-5-1) is an upper-bound for *G*_ST_ whatever the number of alleles.

Let first look for the minimum *H*_s_ at each population. Usually this occurs when one allele is at maximum frequency i.e. 1 giving *H*_s_=0. However in our case the maximum allele frequency is 1 – *maf* and the sum of frequencies can be expressed as

$\sum_{i=1}^{na} p_{i}=\left( 1-maf \right)+\sum_{2}^{na} p_{j}$ with $\sum_{2}^{na} p_{j}=maf$

Therefore

*H*_smin_ = $1-\sum_{i=1}^{na} {p_{i}}^{2}=1- \left( 1-maf \right)^{2}-\sum_{2}^{na} {p_{j}}^{2}$

because $\left( \sum_{j=2}^{na} p_{j} \right)^{2}= \sum_{j=2}^{na} p_{j}^{2}+2\sum_{\begin{aligned} j=2 \\ k>j \end{aligned}}^{na} p_{j}p_{k}$ then we can rewrite

*H*_smin_ = $1-\sum_{i=1}^{na} {p_{i}}^{2}=1- \left( 1-maf \right)^{2}-\left( \sum_{j=2}^{na} p_{j} \right)^{2}+2\sum_{\begin{aligned} j=2 \\ k>j \end{aligned}}^{na} p_{j}p_{k}$

*H*_smin_ = $1- \left( 1-maf \right)^{2}-\left( maf \right)^{2}+2\sum_{\begin{aligned} j=2 \\ k>j \end{aligned}}^{na} p_{j}p_{k}$

The average for *Np* populations (for notational convenience we will use *j* *k* instead *j*=2 with *k*>*j*, in the summatory)

$\bar{H}_{\mathrm{smin}} =1- \left( 1-maf \right)^{2}-\left( maf \right)^{2}+C$

with $C=\frac{2\sum_{j\neq k}^{na} p_{j}p_{k}+2\sum_{j\neq k}^{na} p_{j}^{'}p_{k}^{'}+ \ldots+2\sum_{j\neq k}^{na} p_{j}^{Np-1}p_{k}^{Np-1}}{Np}$

If *na* = 2 then *C* = 0 and

$$\bar{H}_{\mathrm{smin}} =1- \left( 1-maf \right)^{2}-\left( maf \right)^{2}=H_{\mathrm{smin}}^{*}$$

if *na* > 2 then *C* > 0 and obviously $\bar{H}_{\mathrm{smin}}>\bar{H}_{\mathrm{smin}}^{*}$.

Now we are interested in *H*_Tmax_ i.e. the maximum pooled heterozygosity. The maximum *H_t_* occurs when the highest frequency allele at each population is at its maximum i.e. 1-*maf* and there are no shared alleles between populations. Therefore, we have

$\sum_{i=1}^{na} p_{i}=\left( 1-maf \right)+\sum_{2}^{na} p_{j}$ for the first population and

$\sum_{i=1}^{na} p_{i}^{'}=\left( 1-maf \right)+\sum_{2}^{na} p_{j}^{'}$ for the second population and so on if there are more populations.

After pooling we have the sum of frequencies in the whole metapopulation

$$\sum_{i=1}^{na} \frac{p_{i}+p_{i}^{'}+..+ p_{i}^{Np-1}}{Np}=$$

$$=\frac{\left( 1-maf \right)}{Np}+\frac{\left( 1-maf \right)}{Np}+\ldots+\frac{\left( 1-maf \right)}{Np} +\frac{\sum_{2}^{na} p_{j}}{Np}+\frac{\sum_{2}^{na} p_{j}^{'}}{Np}+\ldots+\frac{\sum_{2}^{na} p_{j}^{Np-1}}{Np}$$

Thus noting now *p*_i_/*Np* as the pooled frequency of allele *i*

*H*_t_ = $1- \sum_{i=1}^{Np*na} \frac{p_{i}^{2}}{{Np}^{2}}$ =

$=1-\frac{\left( 1-maf \right)^{2}}{{Np}^{2}}-\ldots-\frac{\left( 1-maf \right)^{2}}{{Np}^{2}}-\frac{\sum_{2}^{na} {p_{j}}^{2}}{{Np}^{2}}-\ldots-\frac{\sum_{2}^{na} {p_{j}^{Np-1}}^{2}}{{Np}^{2}}$ =

$=1-\frac{{Np\left( 1-maf \right)}^{2}}{{Np}^{2}}-\frac{\sum_{2}^{na} {p_{j}}^{2}}{{Np}^{2}}-\ldots-\frac{\sum_{2}^{na} {p_{j}^{Np-1}}^{2}}{{Np}^{2}}=$

$=1-\left( 1-maf \right)^{2}+\left( 1-maf \right)^{2}-\frac{\left( 1-maf \right)^{2}}{Np}-\frac{\sum_{2}^{na} {p_{j}}^{2}}{{Np}^{2}}-\ldots-\frac{\sum_{2}^{na} {p_{j}^{Np-1}}^{2}}{{Np}^{2}}=$

$=1-\left( 1-maf \right)^{2}+\left( 1-maf \right)^{2}(\frac{Np -1}{Np})-\frac{\sum_{2}^{na} {p_{j}}^{2}}{{Np}^{2}}-\ldots-\frac{\sum_{2}^{na} {p_{j}^{Np-1}}^{2}}{{Np}^{2}}=$

and rearranging terms for *maf* in a similar way as we did with *H*_s_ we finally get

$H_{Tmax}=H_{\mathrm{Tmax}}^{*}+\frac{C}{Np}$ (A-5-2)

with

$$H_{Tmax}^{*}=H_{\mathrm{smin}}^{*}+\left( 1-maf \right)^{2}\left( \frac{Np -1}{Np} \right)+\left( maf \right)^{2}\left( \frac{Np -1}{Np} \right)$$

or alternatively, noting that (1 – *maf*)^2^ + *maf*^2^ = 1- *H*^*^_smin_,

$$H_{Tmax}^{*}=\frac{{Np-1+H}_{\mathrm{smin}}^{*}}{Np}$$

and

$$H_{Tmax}=\frac{Np-1+H_{\mathrm{smin}}}{Np}$$

which corresponds to *H*_Tmax_ in equation (4a) in (Hedrick 2005) by taking *K* = *Np* and *H*_s_ = *H*_smin_.

Then, the maximum *G*_ST_ is *G*_STmax_ = 1 – *H*_smin_/*H*_Tmax_. Now we only need to show that

*H*_smin_/*H*_Tmax_ > *H*^*^_smin_/*H*^*^_Tmax_ (A-5-3).

First recall that *H*_smin_ = *H*^*^_smin_ + *C* and similarly *H*_Tmax_ = *H*^*^_Tmax_ + *C/Np* and from the formulae for *H*^*^_Tmax_ in (A-5-2) we appreciate that *H*^*^_smin_ < *H*^*^_Tmax_ so we can express *H*^*^_Tmax_ = *kH*^*^_smin_ with *k*>1. We will proof (A-5-3) by contradiction so let assume that *H*_smin_/*H*_Tmax_ ≤ *H*^*^_smin_/*H*^*^_Tmax_ this implies that (*H*^*^_smin_ + *C*)/ (*kH*^*^_smin_ + *C/Np*) ≤ *H*^*^_smin_/ *kH*^*^_smin_ rearranging terms we get *k* ≤ 1/*Np* which is false. Thus, *H*_smin_/*H*_Tmax_ > *H*^*^_smin_/*H*^*^_Tmax_  and therefore

*G*_STmax_ = 1 – *H*_smin_/*H*_Tmax_ < *G*^*^_STmax_ = 1 – *H*^*^_smin_/*H*^*^_Tmax_

so *G*^*^_STmax_ is an upper bound of *G*_STmax_.

*b) G_STmin_*

It is immediate to show that *G*_STmin_ = 0. Consider a scenario in which every population has the same heterozygosis with the same alleles then *H*_s_ = *H*_T_ and *G*_STmin_ =0 and this is in fact the minimum and the lower bound.

*3) F_STmax_*

For a sequence of biallelic SNPs we will use the *F*_ST_ estimation as defined in (Ferretti, Ramos-Onsins, and Pérez-Enciso 2013) to obtain the upper bound

$$F_{STmax}=\frac{(Np-1)\left[ Np-2\left( 1-maf \right)maf\sum_{k=1}^{Np} \frac{n_{k}}{n_{k}-1} \right]}{Np\left( Np-1 \right)+2\left( 1-maf \right)maf\sum_{k=1}^{Np} \frac{n_{k}}{n_{k}-1}}$$

We proceed as follows; first, we note that the maximum pooled heterozygosity depends on the mean subpopulation heterozygosity *H*_s_. Then we show that both maximum pooled heterozygosity and maximum *F*_ST_ occurs under minimum *H*_s_ and so we compute the minimum for the subpopulation heterozigosity, so that the maximum *F*_ST_ is again *F*_STmax_ = 1 – *H*_smin_/*H*_Tmax_.

In (Ferretti, Ramos-Onsins, and Pérez-Enciso 2013) *H*_T_ is defined as

$$H_{T}=\frac{\bar{H}_{S}}{Np}+\frac{2}{{Np}^{2}}\sum_{k=2}^{Np} \sum_{k^{'}=1}^{k-1} \theta_{\pi a}(k,k^{'})$$

So, for computing *H*_Tmax_ we first seek for the maximum θ_πa_. This maximum will occur when sequences between populations are completely different. Because there are only two alleles and the minimum allele frequency is not 0 but *maf*; the value θ_πa_ computed in this way will be an upper bound and the real maximum would be more or less close to that depending on the relationship between the sample size *n* and the sequence length *L*. In any case this upper bound is valid to ensure an upper bound for *F*_STmax_.

${max\theta}_{\pi a}=\frac{n_{i}n_{j}L}{n_{i}n_{j}L}=1$ for any given pair of populations *i*, *j*. Therefore

$H_{Tmax}=\frac{\bar{H}_{S}}{Np}+\frac{2}{{Np}^{2}}\frac{Np(Np-1)}{2}=\frac{\bar{H}_{S}+Np-1}{Np}$

and

$$F_{ST}=1-\frac{\bar{H}_{S}}{H_{Tmax}}=1-\frac{\bar{H}_{S}}{\frac{\bar{H}_{S}+Np-1}{Np}}=1-\frac{Np\bar{H}_{S}}{\bar{H}_{S}+Np-1}$$

which corresponds to *G*_STmax_ in equation (4a) in (Hedrick 2005) by taking *K* = *Np*.

Furthermore, if we derive *F*_ST_ with respect to *H*_s_ we see that *F*_ST_ decreases with *H*_s_ (the derivative is negative) so the lower the *H*_s_ the higher the *F*_ST_. Consequently, we compute the minimum *H_s_*.

We know that $H_{S}=\frac{\sum_{Np} \theta_{\pi}}{Np}$, where θ_π_ is the mean number of differences between pair of sequences of length *L*. The minimum number of differences at one site will occur when one allele frequency at this site is at the maximum. Obviously, if the allele is at frequency 1 the differences at this site are 0. In our case the maximum frequency allele is (1-*maf*) that in a sample of size *n* implies *n*(1-*maf*) copies of this allele and *n*(*maf*) copies of the alternative, so the number of differences at this site are *n*^2^(1-*maf*)(*maf*) and for *L* sites is *Ln*^2^(1-*maf*)(*maf*). The mean is through *Ln*(*n*-1)/2 pairs so for a given population

$$\theta_{\pi min}=\frac{{Ln}^{2}\left( 1-maf \right)maf}{\frac{Ln(n-1)}{2}}=\frac{2n\left( 1-maf \right)maf}{(n-1)}$$

then for *Np* populations with different sample sizes

$$H_{Smin}=\frac{\sum_{Np} \theta_{\pi min}}{Np}=\frac{2\left( 1-maf \right)maf}{Np}\sum_{k=1}^{Np} \frac{n_{k}}{n_{k}-1}$$

and finally the *F*_ST_ upper bound is

$$F_{STmax}=1-\frac{\bar{H}_{Smin}}{H_{Tmax}}=1-\frac{Np\bar{H}_{Smin}}{\bar{H}_{Smin}+Np-1}$$

by substituting *H*_smin_ and after some rearrangement we get

$$F_{STmax}=\frac{(Np-1)\left[ Np-2\left( 1-maf \right)maf\sum_{k=1}^{Np} \frac{n_{k}}{n_{k}-1} \right]}{Np\left( Np-1 \right)+2\left( 1-maf \right)maf\sum_{k=1}^{Np} \frac{n_{k}}{n_{k}-1}}$$

as an *F*_ST_ upper bound for *Np* populations with different sample sizes in a biallelic setting when the minimum allelic frequency is *maf*.

*4) F_STmin_*

Finally, we show that the lower bound for *F*_ST_ is *F*_STmin_ = 0. Let F_STmin_ = 1 – H_smax_/H_T_.

For simplicity and without loss of generality let assume that the sample size is even in every population. At any site, the maximum number of differences occurs at intermediate allele frequencies and this number is *n*^2^/4 (or (*n*^2^-1)/4 if odd). So, for *L* sites we have *Ln*^2^/4 differences. The mean through *Ln*(*n*-1)/2 pairs for a given population is

$$\theta_{\pi max}=\frac{\frac{{Ln}^{2}}{4}}{\frac{Ln(n-1)}{2}}=\frac{n}{2(n-1)}$$

$$H_{Smax}=\frac{\sum_{Np} \theta_{\pi max}}{Np}=\frac{1}{2Np}\sum_{k=1}^{Np} \frac{n_{k}}{n_{k}-1}$$

which is the same that the *H*_smin_ computation if we substitute *maf* by 0.5.

As we already shown that *F*_ST_ decreases with *H*_s_ we just compute the corresponding pooled heterozygosis when the *H*_s_ is maximum

$$H_{T}=\frac{\bar{H}_{Smax}}{Np}+\frac{2}{{Np}^{2}}\sum_{k=2}^{Np} \sum_{k^{'}=1}^{k-1} \theta_{\pi a}(k,k^{'})$$

Because there are only two alleles and the alleles in any population are at intermediate frequencies, the number of differences in a given site between any pair of populations *i*,*j* is *n*_i_*n*_j_/2 and the average value for *L* sites and pairs of sequences, θ_πa_, is (*Ln*_i_*n*_j_/2)/ *Ln*_i_*n*_j_ = 1/2 for the pair of populations *i*,*j*. So

$$H_{T}=\frac{\bar{H}_{Smax}}{Np}+\frac{(Np-1)}{Np}\frac{1}{2}$$

$$F_{STmin}=1-\frac{\bar{H}_{Smax}}{H_{T}}=1-\frac{\bar{H}_{Smax}}{\frac{\bar{H}_{Smax}}{Np}+\frac{(Np-1)}{Np}\frac{1}{2}}$$

$$F_{STmin}=1-\frac{2Np\bar{H}_{Smax}}{2\bar{H}_{Smax}+Np-1}=\frac{\left( Np-1 \right)(1-2\bar{H}_{Smax})}{2\bar{H}_{Smax}+Np-1}$$

Thus *F*_STmin_ > 0 implies that 1 > 2*H*_smax_ which in turn implies

$$Np>\sum_{k=1}^{Np} \frac{n_{k}}{n_{k}-1}$$

Which is false because *n*_k_/(*n*_k_ -1) > 1 so it follows that *F*_STmin_ ≤ 0. Because we force *F*_ST_ to be 0 the lower-bound will be *F*_STmin_ = 0.

# A-6) Simulations and analysis

*Neutral and selective forward in time simulations*

The simulation design includes a single selective locus model plus one case under a polygenic architecture with 5 selective loci. Two populations of 1000 facultative hermaphrodites were simulated under divergent selection and migration. Each individual consisted of a diploid chromosome of length 1Mb. The contribution of each selective locus to the fitness was 1-*hs* with *h* = 0.5 in the heterozygote or *h* = 1 otherwise (Table B). In the polygenic case the fitness was obtained by multiplying the contribution at each locus. In both populations the most frequent initial allele was the ancestral. The selection coefficient for the ancestral allele was always *s* = 0 while *s* = ± 0.15 for the derived. That is, in population 1 the favored allele was the derived (negative *s*, i.e. contribution 1 + *h*|*s*| in the derived) which was at initial frequency of 10^-3^ while in the other population the favored was the ancestral (positive *s*, i.e. contribution 1 - *h*|*s*| in the derived) and was initially fixed.

**Table B. Fitness Model. The ancestral and derived alleles are noted as *A* and *a*, respectively.**

| **Population** | **Genotypes** | | |
| --- | --- | --- | --- |
|  | ***AA*** | ***Aa*** | ***aa*** |
| **1** | 1 | 1 + \|*s*\|/2 | 1 + \|*s*\| |
| **2** | 1 | 1- \|*s*\|/2 | 1 - \|*s*\| |

|*s*|: absolute value of the selection coefficient.

In the single locus model the selective site was located at different relative positions 0, 0.01, 0.1, 0.25 and 0.5. In the polygenic model the positions of the five sites were 4×10^-6^, 0.2, 0.5, 0.7 and 0.9. Under both architectures, the overall selection pressure corresponded to α = 4*Ns* = 600 with *N* = 1000. Simulations were run in long term scenarios during 5,000 and 10,000 generations and in short-term scenarios during 500 generations. Some extra cases with weaker selection α = 140 (*s* = ± 0.07, *N* = 500) in the long-term (5,000 generations) and stronger selection, α = 6000 (*s* = ± 0.15, *N* = 10,000) in the short-term were also run.

The mating was random within each population. The between population migration was *Nm* = 10 plus some cases with *Nm* = 0 or *Nm* = 50 in a short-term scenario. Recombination ranged from complete linkage between pairs of adjacent SNPs (no recombination, ρ = 0), intermediate values ρ = 4*Nr* = {4, 12, 60} and fully independent SNPs.

A bottleneck-expansion scenario was also studied consisting in a neutral case with equal mutation and recombination rates, θ = ρ = 60, and a reduction to *N* = 10 in one of the populations in the generation 5,000 with the subsequent expansion following a logistic growth with rate 2 and *K*_max_ = 1000.

At the end of each run 50 haplotypes were sampled from each population. For every selective case, 1000 runs of the corresponding neutral model were simulated. To study the false positive rate (FPR) produced by the selection detection tests, the significant results obtained in the neutral cases were counted. The simulations were performed using the last version of the program GenomePop2 (Carvajal-Rodriguez 2008).

In most scenarios, the number of SNPs in the data ranged between 100 and 500 per Mb. However, only the SNPs shared between populations were considered thus giving numbers between 60-300 SNPs per Mb i.e. medium to high density SNP maps.

The interplay between divergent selection, drift and migration (Yeaman and Otto 2011) under the given simulation setting should permit that the adaptive divergence among demes persists despite the homogeneity effects of migration (see *Critical migration threshold* below).

*Map density*

We have seen that *nvdF_ST_* is more sensible to phasing error under lower SNP density (higher pairwise recombination per Mb). In addition, we can also check the effect of using some SNP subsets instead the whole set of shared SNPs.

To perform this experiment we delete some percentage of shared SNPs from the original data set. For example, if we want to evaluate a subset including 90% of the original SNPs we delete 1 SNP out of every 10 from the beginning to the end of the haplotype. Similarly for a subset including 80% from the original we delete 1 out every 5. Finally if we delete 1 out of any two adjacent SNPs we obtain a 50% subset. Of course the linkage relationship between each deleted pair depends on the recombination. This experiment was performed for the same cases as with the phasing accuracy experiment namely ρ = {0, 4, 60}.

The results as appear in Fig C were quite different to the phasing error experiment. The performance in terms of power was not affected. The localization was slightly worse (not shown) by few Kb (a mean localization of 60Kb away when using the complete set becomes 75 Kb away from the real position in the worst case).

The explanation is that for any percentage of deleted SNPs only one adjacent SNP was deleted. Even in the extreme case of deleting 1 SNP out of every 2 the effect is similar to reduce the window size say from 100 to 50 while slightly diminishing the linkage relationship between the markers in the new window size. Therefore the information content within the haplotype pattern was not affected at least under the sample size and evolutionary scenarios evaluated.


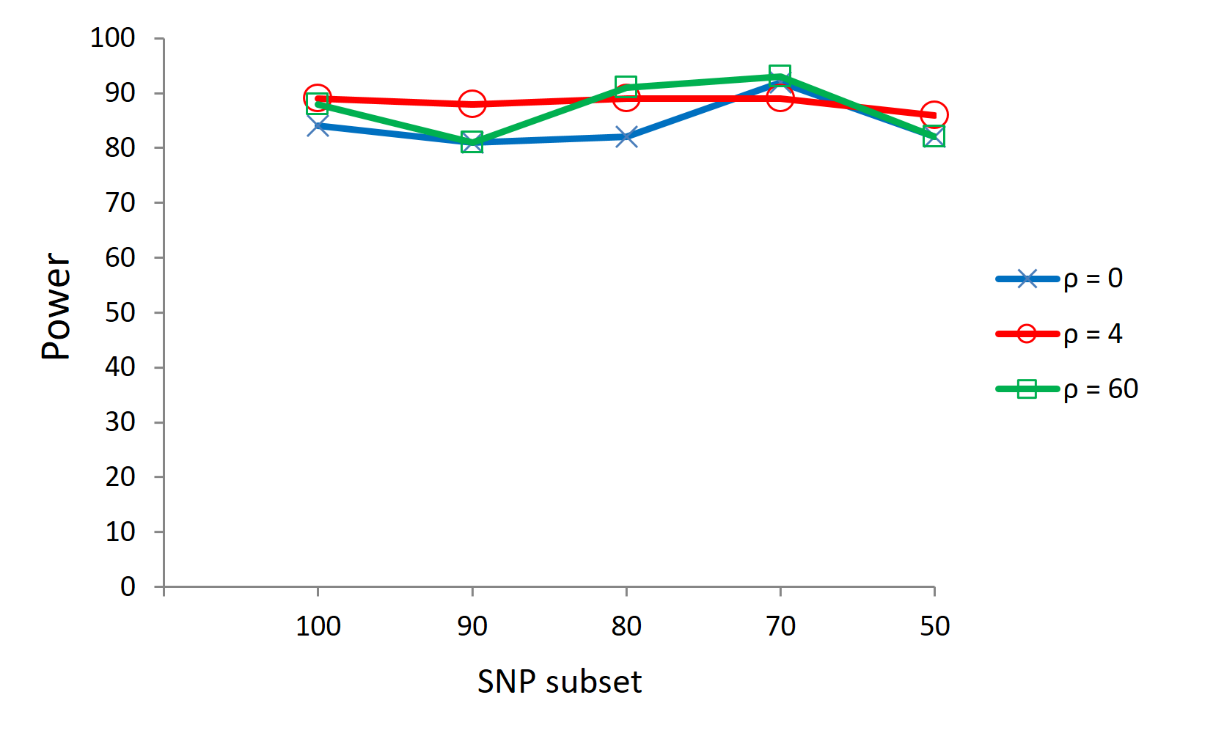


**Fig C. Effect of % SNP subsets on the power of the *nvdF_ST_* test.**

**A-7) Critical migration threshold**

Our simulation model can be viewed as a particular case (with symmetric migration and intermediate dominance) of the model in Yeaman and Otto (2011). These authors develop the model to study the interplay of drift, divergent selection and migration on the maintenance of polymorphism between interconnected populations. They provide a measure, the critical migration threshold, below which adaptive divergence among demes is likely to persist. By rearranging terms in equation (11) from Yeaman and Otto (2011) and after substituting the fitness relationships from our system, we obtain the critical migration threshold for our model:

$m_{crit}=\frac{1}{2}\frac{\left( \frac{\alpha}{2} \right)^{2}-1}{\left( \frac{\alpha}{2} \right)^{2}+4N}$ (A-7-1)

where α = 4*Ns* . For each selective pressure, we can therefore compute the critical number of migrants (*Nm*_crit_) below which the selective polymorphism should be present in the data. The weaker the selection the lower the threshold so, for α = 140 the minimum critical number of migrants is 177 individuals. Thus, our highest migration *Nm* = 50 is below the threshold. This means that both scenarios *Nm* = 10 and 50, would permit to maintain the locally adaptive allele for every selective scenario assayed (weak, intermediate and strong) despite the homogeneity effects of migration.

# Bibliography

Carvajal-Rodriguez, A. 2008. GENOMEPOP: A program to simulate genomes in populations. BMC Bioinformatics **9**:223.

Charlesworth, B., and D. Charlesworth. 2010. Elements of evolutionary genetics. Roberts and Company Publishers, Greenwood Village, Colo.

Ferretti, L., S. E. Ramos-Onsins, and M. Pérez-Enciso. 2013. Population genomics from pool sequencing. Molecular Ecology **22**:5561-5576.

Friguet, C. 2012. A general approach to account for dependence in large-scale multiple testing. Journal de la Societé Francaise de Statistique **153**:100-122.

Hedrick, P. W. 2005. A standardized genetic differentiation measure. Evolution **59**:1633-1638.

Hudson, R. R. 2002. Generating samples under a Wright-Fisher neutral model of genetic variation. Bioinformatics **18**:337-338.

Hussin, J., P. Nadeau, J.-F. Lefebvre, and D. Labuda. 2010. Haplotype allelic classes for detecting ongoing positive selection. BMC Bioinformatics **11**:65.

Meinshausen, N., and J. Rice. 2006. Estimating the Proportion of False Null Hypotheses among a Large Number of Independently Tested Hypotheses. The Annals of Statistics **34**:373.

Nei, M. 1973. Analysis of gene diversity in subdivided populations. Proceedings of the National Academy of Sciences **70**:3321-3323.

Rivas, M. J., S. Dominguez-Garcia, and A. Carvajal-Rodriguez. 2015. Detecting the Genomic Signature of Divergent Selection in Presence of Gene Flow. Current Genomics **16**:203-212.

Sharma, R., M. Gupta, and G. Kapoor. 2010. Some better bounds on the variance with applications. Journal of Mathematical Inequalities **4**:355-363.

Storey, J. D. 2002. A Direct Approach to False Discovery Rates. Journal of the Royal Statistical Society. Series B (Statistical Methodology) **64**:479.

Storey, J. D. 2001. Estimating false discovery rates under dependence, with applications to DNA microarrays.

Storey, J. D., J. E. Taylor, and D. Siegmund. 2004. Strong control, conservative point estimation and simultaneous conservative consistency of false discovery rates: a unified approach. Journal of the Royal Statistical Society Series B-Statistical Methodology **66**:187-205.

Storey, J. D., and R. Tibshirani. 2003. Statistical significance for genomewide studies. Proc Natl Acad Sci U S A **100**:9440-9445.

Yeaman, S., and S. P. Otto. 2011. Establishment and Maintenance of Adaptive Genetic Divergence under Migration, Selection, and Drift. Evolution **65**:2123-2129.
