# Supplementary material for: HacDivSel: Two new methods (haplotype-based and outlier-based) for the detection of divergent selection in pairs of populations
Source: PLoS One. 2017 Apr 19;12(4):e0175944. doi: 10.1371/journal.pone.0175944 (PMC5397020; doi:10.1371/journal.pone.0175944)
Supplement: S1 Program Manual — Description: Complete manual for the program that implements the methods described in the paper. (PDF) [file pone.0175944.s003.pdf]

HacDivSel Manual v 1.3

Antonio Carvajal-Rodríguez

Facultad de Biología, Campus Lagoas-Marcosende

Departamento de Bioquímica Genética e Inmunología

Universidad de Vigo, Vigo 36310, Spain

Email: acraaj@uvigo.es

Web: http://acraaj.webs.uvigo.es

TABLE OF CONTENTS

Introduction 3
The Progam 3
Sliding window with full overlapping 3
Non full window overlapping 4
Download 5
Ready-to-use executables 5
Compiling the program manually (linux/MAC) 5
Input 5
MS format 6
FASTA format 7
HapMap phased format 7
Genepop format 8
BayeScan format 8
PLINK flat files (MAP/PED) format 8
MAP files 8
PED files 9
Run the program 10
Command line options 10
Default Values 12
Minimum allele frequency 12
Run in MacOsX or Linux 13
Run in Windows 13
Output 13
References 15

INTRODUCTION

HacDivSel is a program for the detection of divergent selection in pairs of populations connected by migration. It includes a new model, called normalized variance difference nvd, from the preceding Svd (Hussin *et al.* 2010) and SvdM (Rivas *et al.* 2015) statistics. The statistic is computed for haplotypes coming from a pair of populations and analyzes every SNP under various window sizes. The method is defined for reaching higher power when the selected allele frequency is 0.5 in the pooled populations. At different window sizes, the SNPs giving the maximum value are selected as candidates and then, an F_{ST} index comparing the overall F_{ST} with the F_{ST} at each candidate site is computed. The *p*-values are obtained by resampling the data using the mean SNP frequencies calculated by pooling the two populations. For the case when the markers are unlinked (no haplotypes), the program also incorporates a new extreme-outlier set (EOS) test. See (Carvajal-Rodriguez, 2017) for details on the methods.

THE PROGAM

Sliding window with full overlapping

Full overlapping is the default option. Under this setting every SNP is inspected for computing its haplotype allelic class (HAC) value. The program works in one of two modes: fixed or variable window size. The latter is the default mode, where an automatic search through different decreasing windows, is done. For a given window size L each inspected SNP is intended to be positioned at the center of the window. Consider the range 0 to (L-1) with center C = (L-1)/2. Now if we want to put a SNP position p at the center, the initial and ending positions of the window will be

$$ini = p - C$$

$$end = p + C = ini + L$$

with the window being the interval [ini, end). So, while the SNP is at the left of the first C positions in the haplotype ($p < C$) it cannot be centered and then the same region ini =0 end = L is used in those cases. Similarly, if the ending extreme of the window is located beyond the number N of SNPs ($p > N - C$) then $ini = N - L$, $end = N$ is used. As indicated, the program works in automatic (i.e. variable) mode by default. This can be changed to a fixed window size, by the argument –*window <window size>* e.g. –*window* 100 sets a fixed size of L = 100. The automatic, default, mode is equivalent to use –*window* 0. In this case, the program sets initially $L_0 = min(N, MaxWin)$ where N is the number of SNPs in the sample

and *MaxWin* is the user defined maximum window size (1,000 by default). Under the variable window mode, a number of iterations are successively performed dividing L by a window reduction factor (2 by default) each time. The number of iterations is computed as

$$min\{floor(log(L_0/minWinSize)/log(WinReductFactor)) + 1 , MaxWinIter\}$$

where *MaxWinIter* is by default 5. The maximum number of iterations and the window division factor can also be defined by the user (see *command line options*, below).
Obviously, the higher the window size the slower the computation. For example, it took almost three hours to solve a window size of 90,000 SNPs working in a 64 bit Intel i3 with 1.8 GHz and 8 GB RAM under Windows 8.1 but about 15 minutes for the same file, when the windows size was fixed to 1,000 or under the variable mode (recall that the default maximum window size under this mode is 1,000) just to get almost exactly the same result (p value 0.64 vs. 0.69).
Additionally, to afford computation time, when the number of SNPs is above 10,000 the number of resamplings is automatically down to 100 instead of the default 500. This is justified because after performing several simulations, the results are virtually identical using 100 or 500 resamplings. The higher number is being used by default because it warrants a more precise estimate of the p-values.

The allowed minimum window size is by default 51. Thus if the data contains less than 51 SNPs the program skip that file. This value can also be changed by the user.

Non full window overlapping

HacDivSel may also work under a non-overlapping model. To define partial or non-overlapping modes use the argument –*overlap* 1. This implies that for each window size L, the first *SNP* to inspect is at position (L - 1)/2 (the center of the window) and the next inspected SNP will be after L / *nolf* positions. The value of the non-overlapping (*nolf*) factor is by default 2 (the minimum is 1). The user may decide to change it by the argument –*overlapfactor<nolf>*. Therefore -*overlapfactor* 1 proceeds from the center of one window to the center of the adjacent window without overlapping i.e. it checks only one SNP (the central one) by window. Similarly -*overlapfactor* 2 proceeds from the center of one window to the next L/2 positions SNP, so it checks two SNPs by window. The maximum value for the factor is L. In this case the next position is L/L = 1 and we recover the full overlapping case, with just one difference, because the first SNP assayed continue to be located at the center of the first window (position (L - 1)/2) while in the default case, –*overlap* 0, the first SNP assayed is the first one (position 0).

Although full overlapping is the default program mode, the partial overlapping model is enforced automatically when the number of SNPs is more than one million. In this case (more than 1E6 SNPS and the user did not define –*overlap* 1), the system automatically changes to

–*overlap* 1 –*maxwin* 10000 –*winfactor* 10

The rationale under these changes is that with so many SNPs the density is higher and so the linkage information should remain in larger windows. If the user has million SNPs but prefer other settings she/he just needs to define –*overlap* 1 which prevents any automatic change by the program.

If a non-overlapping factor is applied (user defined or automatic) the tag "_noL<*nolf*>" is added to the output file name.

Download

The program can be downloaded from

http://acraaj.webs.uvigo.es/software/HacDivSel.zip

Ready-to-use executables

Windows 64 bits: HDSel1.3w64.exe

Ubuntu: HDSel1.3U64

Compiling the program manually (linux/MAC)

Go to the source folder that should include a file called Makefile, and just type make.

INPUT

The acceptable formats for the input file are MS, Fasta, HapMap phased, Genepop, BayeScan (codominant markers) and PLINK. The three last formats allowing only for the execution of the outlier-based method.

For any of these formats HacDivSel accepts as commentaries those lines in the very initial part of the file beginning with #.

Additionally, under the formats Genepop, HapMap and PLINK, any line in any part of the file can be a commentary if it begins with #. Therefore, for these files we can comment some lines in order to eliminate specific individuals (Genepop and PLINK-ped) or SNPs (HapMap) from the sample.

MS format

The MS format is the standard Hudson's ms output format. For example:

```
ms 2 2 -t 60
104642148

//
segsites: 3
positions: 0.002771	0.004018	0.014038
000
111

//
segsites: 3
positions: 0.1	0.4	0.8
010
101
```

To use this format (is the default so no argument is needed at all):

–format ms.

We can read two runs from the same file as if they were different populations (the example above) as produced in the output of GenomePop software for samples of two subpopulations. In this case the sample size is assumed to be the same for both populations. For example, if we have generated the file called mu_r_60Nm_10.txt that has 50 haplotypes sampled from each population; to analyze it under HacDivSel we type:

./HDSel -path ./ -input mu_r_60Nm_10.txt -candidates 1 -output test.out -sample 50 -format ms -maf 4

Alternatively, we could has just one run in the file that is a mixture of two populations as produced by using the corresponding arguments when executing the MS program

./ms 100 1 -t 60 -r 60 1000000 -I 2 55 45 40 >> mu_r_60Nm_10.txt

that produces one run with a sample of 100 sequences: 55 coming from one population and 45 from another. Then we get, for example:

```
ms 100 1 -t 60 -r 60 1000000 -I 2 55 45 40
104642148

//
segsites: 300
positions: 0.002771	0.004018	0.014038 ...
000 ...
```

111 ...
110 ...
010 ...
.
.
000 ...

In this case if we want that HacDivSel reads the file we should indicate both sample sizes

./HDSel -path ./ -input mu_r_60Nm_10.txt -output test2.out -sample 55 -sample2 45 -format ms -maf 4

FASTA format

The program reads the FASTA sequential format. It allows symbols A, C, G and T. Entries with any other symbol represent unresolved nucleotides and are treated as null alleles. The number of populations is computed from the total number of sequences dividing by the user-given sample size. It assumes equal sample size. When preparing the data for the computation, the following parsing is performed. In the reference population (the first, by default under equal sample sizes), the most frequent allele at each position will be noted as '0' and any alternate as '1'. If the most frequent symbol is N that site is ignored. Thus, if the most frequent allele in the reference population is, say, T then this base will be noted as '0' and any other as '1' for this site in the whole data set (all populations). To use this format:

–format fasta

So, with the same example as above, having a file called mu_r_60Nm_10.fasta we call

./HDSel -path ./ -input mu_r_60Nm_10.fasta -candidates 1 -output test.out -sample 50 -format fasta -maf 4

HapMap phased format

Files are organized in a *SNPs x haplotypes* format, so each row represents a SNP and each column a haplotype. The first row (header row) contains the identifiers of the individuals that have been phased. The first column (header column) contains the rsID of the SNPs. The second column contains the physical position of these SNPs in the particular chromosome. Entries with anything other than A, C, G, or T represent unresolved nucleotides and are treated as null alleles. To use this format:

–format hm

The program expect two files, one for each population (can have different sample sizes). To make the program read one population from each file we need to add the argument –

singlepopfile 1. For example, to analyze the files *hm3_chr2_ceu.txt* and *hm3_chr2_yri.txt* that are in a folder called HapMap, we use the following arguments:

./HDSel -path ./HapMap/ -input hm3_chr2_ceu.txt -input2 hm3_chr2_yri.txt -candidates 0.1% -output hmCEUvsYRI -format hm -singlepopfile 1

where from the SNPs shared between the two populations, the 0.1% of the best candidates at each assayed window size will be tested.

Genepop format

This format has no haplotype information thus, only the EOS test is performed using the G_{ST} estimator. It allows variable sample size as inferred from the data.
To use this format:

–format gp

./HDSel -path ./ -input mu_r_60Nm_10.genepop -output test.out -format gp -maf 4

BayeScan format

This format has no haplotype information thus, only the EOS test is performed using the G_{ST} estimator. The version of the data format is for codominant markers. It assumes equal sample size in both populations. For example if the number of copies is 2N = 100 we set -*sample* 50. To use this format:

–format bs

./HDSel -path ./ -input mu_r_60Nm_10.bs -output test.out -format bs -sample 50 -maf 4

PLINK flat files (MAP/PED) format

This format has no haplotype information thus, only the EOS test is performed using the G_{ST} estimator. It allows variable sample size which is inferred from the data.

MAP files

The fields (separated by space or tab) in a MAP file are:

- Chromosome
- Marker ID

- Genetic distance
- Physical position

For example:

```
2	rs11901199	0	8856
2	rs7594567	0	11833
```

The column with the genetic distances is optional. The following example is also valid:

```
2	rs11901199	8856
2	rs7594567	11833
```

Thus, the number of columns must be 4 or 3, a mixture of both is not valid.

PED files

The fields (separated by space or tab) in a PED file are

- Population ID
- Individual ID
- Paternal ID
- Maternal ID
- Sex (1=male; 2=female; other=unknown)
- Phenotype (0=unknown; 1=unaffected; 2=affected)
- Genotypes (space or tab separated, 2 for each marker)

For example, corresponding to the map file above, the first line could be:

```
Pop1	NA19176		0 0 1 1	G G C C
…
```

Note that the length of the genotype must be 2x the number of data rows in the MAP file.

The genotypes can be nucleotides or numbers. In the case of nucleotides there are only 4 alleles (A, C, G or T) and any other symbol will be considered as missing information (N).

To use this format with nucleotides:

–format pl

In the case that genotypes are numbers, the maximum number of alleles allowed in the current version is 255 and 0 is the symbol for a missing allele.
To use this format with numbers:

–format pl2

In any case, if the name of map and ped files is the same e.g. *mu_r_60Nm_10*.map and *mu_r_60Nm_10*.ped then the input file for the program must be

-input mu_r_60Nm_10

without the extension.

For example, for nucleotides:

./HDSel -path ./ -input mu_r_60Nm_10 -output test.out -format pl -maf 4

Or for numbers:

./HDSel -path ./ -input mu_r_60Nm_10 -output test.out -format pl2 -maf 4

That read both the map and ped files. However, if the names of map and ped files are different e.g. *mu_r_60Nm_10*.map and *mu_r_60Nm_10*_nuc.ped then the input for the program must be

*-input mu_r_60Nm_10 -input2 mu_r_60Nm_10*_nuc *-singlepopfile 1*

again without the extension and with the flag *singlepopfile* to indicate that two different file names are being considered. Note that in this latter case the *-input2* name must be always the ped file name. For example, with nucleotides:

./HDSel -path ./ -input mu_r_60Nm_10 -input2 mu_r_60Nm_10_nuc -singlepopfile 1 -format pl -maf 4

RUN THE PROGRAM

Command line options

The following arguments can be passed to the program:

-candidates <INTEGER> The number of candidates to look for selection using the $nvdF_{ST}$ method. By default is 1. If the number is followed by % then the given number is the percentage over the analyzed SNPs.

- cutoff<DOUBLE> Define the value *numdesvs* in the computation of the cutoff for the EOS test (cutoff = fsttot+numdesvs*maxdev). Default is 1.25.

-help|-h Displays this help message.

-input <STRING> Specifies the name of the input file.

-input2 <STRING> Specifies the name of the second input file. It is necessary only when the argument -singlepopfile is set to 1.

-format <STRING> Specifies the sequence data format (BS, Fasta, GP, HM, PL or by default MS).

-maf <DOUBLE> Defines the absolute minimum allele number in the metapopulation (default is 4).

-maxiter <INTEGER> Defines the maximum number of different window sizes (default is 5) to try under the variable window size setting.

-maxwin <INTEGER> Defines the maximum window size (default is 1000) under the variable window size setting. If set to 0 then it defines all available SNPs as the maximum window size.

-minwin <INTEGER> Defines the minimum window size (default is 51 SNPs).

- onlyEOS < INTEGER > Defines if only the EOS test is performed. Default is 0 ($nvdF_{ST}$ and EOS performed). It is automatically set to 1 when the format is BS, GP or PL.

- onlyrsidInfo <INTEGER> By default is 0. When set to 1 the program generates an output file with the names, positions and frequencies of the whole set of analyzed SNPs.

-output <STRING> Specifies the header for the name of the output file.

-overlap <INTEGER> By default is 0 which implies full overlapping. If set to 1 a non-overlapping factor (default value 2) is applied.

-overlapfactor <INTEGER> Requires that the -overlap argument is defined to 1. By default the value of the overlapping factor is 2.

-path <STRING> Specifies the path to the input file.

-sample <INTEGER> Defines the number of alignments in each population sample (50 by default). If under the ms format we want to read two populations with different sample sizes we need to add a second argument –sample2 (see below). Under the GenePop format the sample size is allowed to vary between populations. Under the BayeScan format the size is implicitly allowed to vary (total number of genes / ploidy) but the value *sample* will be used to compute the minimum allele frequency. Under the HM format the two files can have different sample sizes that will be recognized automatically.

-sample2 <INTEGER> Defines the number of alignments in a second population sample under the MS format. By default has value 0, in this case the program reads two populations (marked with //) from one MS file and expects that each population has the same sample size. If sample2 has a positive value then the program expects

a second input file name (-input2 …) and would read each population in MS format from a different file: *-path ./msfiles/ -input msfile1 -input2 msfile2 -candidates 1% -output testmsf -sample 40 -sample2 55 -format ms*

-singlepopfile <INTEGER> This is only necessary under the HM or PLINK formats. By default is 0 so it is assumed that there are two populations (with equal sample size) at each HM input file. When set to 1 this means that two different filenames (-input and -input2, one for each population) should be provided for the program to work. In the case of PLINK this is used for reading map and ped files with different names (see PLINK format section).

-SL <DOUBLE> Defines the uncorrected significance level (γ = 0.05 by default).

-verbose<INTEGER> Provides additional information during the computation and a log file at the end.

-window <INTEGER> Defines the fixed window size. If 0 the window size will be variable (this is the option by default).

-winfactor <INTEGER> Defines the factor that divides the window size each new iteration (default is 2) under the variable window size setting.

Default Values

Calling the program without arguments, i.e.

./HDSel

It is equivalent to calling

./HDSel -path ./ -format ms -input hacdivselF –input2 hacdivselF -window 0 -sample 50 –sample2 0 -maf 4 -candidates 1 -cutoff 1.25 -output _maxMVD_wsVariable.xls -SL 0.05 -minwin 51 -maxwin 1000 -maxiter 5 -overlap 0 –overlapfactor 2 -onlyEOS 0 -winfactor 2 –singlepopfile 0 - onlyrsidInfo 0 –pref 1 –verbose 0

Minimum allele frequency

The $nvdF_{ST}$ method only considers the SNPs shared between populations. Thus, if a SNP is not shared is eliminated independently of its frequency. For the shared SNPs, we consider alleles at a given frequency, for example if MAF = 4 (default value) then the minimum frequency should be more than two copies (4 alleles/ 2 populations) by population (if sample sizes are equal). In general, the minimum frequency at each population will be MAF/ sum of sample sizes.

Run in MacOsX or Linux

If the downloaded executable has name HDSel (can vary for different versions), from the console or terminal just type ./HDSel jointly with the desired arguments.

Run in Windows

Double click just for running the program under default options. Alternatively, you can go to the command prompt (cmd.exe) and type

HDSel

You can also access the Run command by pressing the Windows logo key +r
then drag and drop the .exe file from your folder and add the desired arguments, e.g. if the HDSel.exe is in the folder HACDivSel then after drag and drop you will have
C:\HACDivSel\HDSel.exe
now add the desired arguments and then hit the Intro key. For example:
C:\HACDivSel\HDSel.exe –input neutral.txt –output neutralres

OUTPUT

If the *ouput* tag in the argument list was followed by *neutralres* (-output neutralres) then the output generates one file called *neutralres _maxMVD_wsVariable.xls* if the variable window mode was used or *neutralres_maxMVD_wsConst51.xls* if a window size of 51 was fixed. In any case the file would contain the following columns if the $nvdF_{ST}$ method was performed:

File: The name of the analyzed file.
Sample size: Give the sample size in the case this is constant or the minimum analyzed sample size in the case it varies (hm format).
rsid (only under hm format): The rsID of the given SNP.
SNP: Location corresponding to the candidate having the maximum *nvd* value.
IniPos: Location for the beginning of the window size.
EndPos: Location for the end of the window size.
Candidate: The candidate number if more than one were defined.
Wide: The window size in number of SNPs.
nvd: The nvd value corresponding to the SNP candidate.
sgnTest: The value computed from (5) in (Carvajal-Rodriguez 2017).
FST: The F_{ST} value at the candidate site.

FSTIndex: For a given candidate *i* the F_{ST_i} minus the overall F_{ST} value.
***D'*:** Average linkage disequilibrium for the window size used to compute a given candidate.
Meff: Estimate of the number of independent SNPs between the most distant selected candidates.
Pval: The *p*-value computed by resampling the data using the mean of the two population frequencies weighted by each population sample size.
Sig: Indicates the significance: ** if (1) and (2) are true; *: only (1) is true.
(1): The *p*-value was lower than the corrected significance level $\alpha = 1 - (1 - \gamma)^{1/C}$ where $C = max\{(\theta_{\pi max} - \theta_{\pi})/(\theta_{\pi max} - \theta_{\pi min})minwin, (candidates + \text{WinIter})\}$. θ_{π}: average number of differences between pairs of sequences computed for a given window size. If sample size *n* is even then $\theta_{\pi max} = n/(2*(n-1))$ and $\theta_{\pi min} = 2*maf*(n-maf) / [n*(n-1)]\ maf$.
(2): The sign test was positive.
In any case the users can perform any desired correction by considering the *p*–value, the number of independent SNPs between candidates and/or the total SNPs (the #SNPs item below).
Qval: The *q*-value corresponding to the given *p*-value.

Additionally, the output file presents the results of the extreme outlier set (EOS) test, firstly indicating the value of MAF applied, the percentage of files (if any) having extreme outliers (under just 1 file it could be only 0 or 100%) and then, the following columns:
File: The name of the analyzed file.
FstUB: The F_{ST} or G_{ST} upper bound for that file.
Fstmean: The F_{ST} or G_{ST} mean for the analyzed loci.
#SNPs: Total number of analyzed SNPs.
#indepSNPS: Estimated number of independent SNPs.
#Outliers: The total number of outliers detected for this file.
#Eoutliers: The number of outliers in the extreme outlier set.
PI0: The (a priori) estimated proportion of true nulls for the *q*-value calculation.
LBq: Lower bound for the *q*-value.
Name: Name of the locus for this extreme outlier.
SNP: The location of the outlier.
FST: The F_{ST} or G_{ST} value.
P: The *p*-value after the LK test. A multiple testing correction can be applied by the user for the number of outliers in the extreme outliers set (*Eoutliers*).
qval: The estimated *q*-value.
q': The corrected *q*-value, $q' = (\text{qval} - \text{LBq}) / (1 - \text{LBq})$.

Finally a second file is written with the same name plus _FST as a postfix e.g. *neutralres _maxMVD_wsVariable_FST.xls* containing the whole distribution of outliers.

REFERENCES

Hussin J, Nadeau P, Lefebvre J-F, Labuda D (2010) Haplotype allelic classes for detecting ongoing positive selection. *BMC Bioinformatics* **11**, 65.

Rivas MJ, Dominguez-Garcia S, Carvajal-Rodriguez A (2015) Detecting the Genomic Signature of Divergent Selection in Presence of Gene Flow. *Current Genomics* **16**, 203-212.

Carvajal-Rodriguez (2017) HacDivSel: Two new methods (haplotype-based and outlier-based) for the detection of divergent selection in pairs of populations.
